# Potential risk factors for jaw osteoradionecrosis after radiotherapy for head and neck cancer

**DOI:** 10.1186/s13014-016-0679-6

**Published:** 2016-07-30

**Authors:** Thomas Kuhnt, Andreas Stang, Andreas Wienke, Dirk Vordermark, Ramona Schweyen, Jeremias Hey

**Affiliations:** 1 Department of Diagnostic Imaging and Radiation Medicine, University Clinic, University Leipzig, Leipzig, Germany; 2 Department of Medical Informatics, Biometry and Epidemiology, University Clinic Essen, Essen, Germany; 3 Department of Medical Epidemiology, Biometry and Computer Science Martin-Luther-University Halle-Wittenberg, Halle, Germany; 4 Department of Radiotherapy, University Clinic, Martin-Luther-University Halle-Wittenberg, Halle, Germany; 5 Department of Prosthetic Dentistry, University School of Dental Medicine, Martin-Luther-University Halle-Wittenberg, Halle, Germany

**Keywords:** Osteoradionecrosis, Radiotherapy, Head and neck cancer, Dental status, Bone surgery, Tumor site

## Abstract

**Introduction:**

To identify potential risk factors for the development of jaw osteoradionecrosis (ORN) after 3D-conformal radiotherapy (3D-CRT) and intensity-modulated radiotherapy (IMRT) among patients with newly diagnosed head and neck cancer.

**Material and methods:**

This study included 776 patients who underwent 3D-CRT or IMRT for head and neck cancer at the Department of Radiotherapy at the University Hospital Halle-Wittenberg between 2003 and 2013. Sex, dental status prior to radiotherapy, tumor site, bone surgery during tumor resection, concomitant chemotherapy, and the development of advanced ORN were documented for each patient. ORN was classified as grade 3, 4, or 5 according to the Radiation Therapy Oncology Group/European Organization for Research and Treatment of Cancer classification or grade 3 or 4 according to the late effects in normal tissues/subjective, objective, management, and analytic scale. The cumulative incidence of ORN was estimated. Cox regression analysis was used to identify prognostic risk factors for the development of ORN.

**Results:**

Fifty-one patients developed advanced ORN (relative frequency 6.6 %, cumulative incidence 12.4 %). The highest risk was found in patients who had undergone primary bone surgery during tumor resection (hazard ratio [HR] = 5.87; 95 % confidence interval [CI]: 3.09–11.19) and in patients with tumors located in the oral cavity (HR = 4.69; 95 % CI: 1.33–16.52). Sex, dentition (dentulous vs. edentulous), and chemotherapy had no clinically relevant influence.

**Discussion and conclusion:**

In contrast to most previous studies, we noted a low cumulative incidence of advanced ORN. Patients with tumors located in the oral cavity and those who undergo bone surgery during tumor resection prior to RT may be considered a high-risk group for the development of ORN.

## Background

During the last decade, there have been several technical advancements in radiation therapy (RT) that reduce acute and chronic therapy-related side effects, especially in patients undergoing RT for head and neck cancer [1]. The introduction of three-dimensional conformal RT (3D-CRT) and intensity modulated RT (IMRT) allows for greater protection of the major salivary glands thus preserving the salivary flow rate and allowing better recovery of the salivary glands [2, 3]. Consequently, patients’ quality of life has been improved, and the risk of radiation-induced damage to dentition has decreased [4].

Currently, osteoradionecrosis (ORN) of the jaw is one of the most severe chronic side effects of RT to the head and neck region [1]. The associated morbidity of this condition and its subsequent treatment, which can range from close observation to radical surgical resection, can be substantial [5–7].

According to the current theory of Delanian and Lefaix [8], ORN is irradiation-induced fibrosis with histopathological formation phases very similar to those of chronic wounds. The key event in the development of ORN is the activation and regulatory disturbance of fibroblast activity. The combination of dying osteoblasts without osteoblast replication and excessive proliferation of myofibroblasts results in a reduction of bone structure. The regulatory disturbance leads finally to vulnerable, atrophic-fibrous tissue in the irradiated area. The irradiation dose to the bone is believed to be associated with the risk of ORN. However, the mandible seems to be especially susceptible to the development of ORN because the blood supply is limited to a single functional terminal artery. The facial artery does not seem to be able to produce enough collateral blood vessels to compensate for the loss of the blood supply to the mandible that occurs after fibrosis of the inferior alveolar artery.

In addition, with the optimized irradiation techniques, the irradiation dose to the mandible differs considerably depending on tumor site. For example, in cases of tumors that are located within the oral cavity, the irradiation dose to the mandible is usually high [9–12].

In addition to the irradiation dose, other factors have been reported to increase the risk of ORN. In one study, a sex-depended risk was detected [10]. Women were found to have a significantly lower risk of ORN development. The three times higher relative frequency of ORN among men was considered to be attributed to their higher nicotine consumption.

Concomitant chemotherapy may also be a potential risk factor for ORN development. Cisplatin derivatives are the most commonly used chemotherapeutic drugs; the function of these derivatives is based on the intracellular generation of an increased number of free oxygen radicals, so-called as reactive oxygen species, which inhibit the DNA repair capacity of the bone cells in the normal tissues [13]. According to the theory of Delanian and Lefaix, this might have a major initial impact on the pathomechanism of ORN development [8]. Furthermore, bone surgery immediately preceding RT may influence the risk of ORN development. Monnier et al. showed that among patients with ORN, 92 % required bone surgery of the mandible due to tumor resection prior to RT [12]. In addition, affected teeth can act as an entry point for pathogenic germs that may influence the development of ORN [14]. Most studies on risk factors for ORN development were conducted decades ago. With the considerable modernization of irradiation techniques during the last 10 years, a new evaluation of the potential risk factors for ORN development, including sex, tumor site, bone surgery, chemotherapy, and dentition may give new insight into the etiology of ORN.

## Material and methods

Patients who underwent high-dose RT for head and neck cancer between January 1, 2003, and January 31, 2013, at the Department for Radiotherapy at the University Hospital of Halle-Wittenberg were included. Inclusion criteria were primary tumors in the nasopharynx, oropharynx, uvula, tongue base, oral cavity, parotid gland, or larynx/hypopharynx. The protocols were approved by the medical faculty’s ethics committee at the Martin-Luther-University Halle-Wittenberg and conducted in accordance with the Declaration of Helsinki on Ethical Principles for Medical Research.

### Surgery

Tumor and neck lymph node removal was performed in patients with early or locally advanced tumors who were in good general condition.

### Bone surgery during tumor resection

Bone surgery was defined as a risk factor if removal of the jaw bone was necessary during tumor resection. This also included tumor operations that involved temporary splitting of the mandible in order to gain access to the pterygopalatine fossa, the parapharyngeal space, and the oropharynx [15, 16]. According to the investigation of Studer et al. surgical interventions were classified [17]. In addition to periostal stripping, marginal resection and segmental resection a fourth group, temporary splitting of the mandible, was implemented.

### RT

Three-dimensional treatment planning with 3D-CRT (from 2003 to 2013) or IMRT (from 2006 to 2013) was performed in all patients. Treatment planning was based on a computed tomography (CT) scan of the head and neck region, with a slice thickness of 5 mm (Lightspeed; General Electric, Fairfield, USA). Patients were immobilized using a custom-made thermoplastic head–neck–shoulder mask. Two planning systems (Helax TMS version 6.1 and Oncentra Masterplan version 1.5/3.0; Nucletron, Veenendaal, Netherlands) were used for the 3D treatment planning. 3D-CRT was performed using standardized six to seven portal arrangements as described previously [18]. Patients receiving 3D-CRT were treated with 6- and 10-MV photons from a linear accelerator (Primus and Oncor; Siemens Medical Solutions, Erlangen, Germany). IMRT was based on the step-and-shoot approach with seven or nine equidistant 6-MV beams and five to 8 subsegments, respectively. The treatment technique was similar to the one described by Georg et al. [19]. The planning strategy was to cover 95 % of the planning target volume (PTV) with 95 % of the prescribed dose. The mean dose given to at least one parotid gland was limited to 26 Gy without compromising the PTV. The maximum dose to the spinal cord was 45 Gy. Irradiation planning was performed according to reports 50 and 62 of the Commission on Radiation Units and Measurements (ICRU) [20, 21]. Planning, performance, and quality assurance were undertaken according to ICRU report 83 [22].

- *Postoperative RT:*
  The fractionation schedule was the traditional 2.0 Gy/day, 5 days a week. A total dose of 64 to 70 Gy was delivered for each patient.
- *Definitive RT:*
  Patients received hyperfractionated-accelerated RT with 70.6/77.6 Gy in 15 fractions of 2 Gy followed by 1.4 Gy twice a day or with 72 Gy in 14 fractions of 1.8 Gy followed by 1.8 Gy and 1.6 Gy twice daily.

### Chemotherapy

Some patients received postoperative adjuvant or definitive radiochemotherapy. The indication for chemotherapy was determined by a specialist in RT in the University Clinic Halle-Wittenberg, who also then prescribed a regimen:

- *Concomitant adjuvant chemotherapy with standard fractional RT:*
  Cisplatin (20 mg/m^2^/day as a 30-min infusion) administered on days 1–5 and 29–33 of RT
- *Concomitant definitive chemotherapy with hyperfractionated-accelerated RT:*
  Cisplatin (40 mg/m^2^/day as a 30-min infusion) administered on days 1, 8, 15, 22, and 29 of RT
- *Concomitant adjuvant chemotherapy with standard fractional RT:*
  Cisplatin (20 mg/m^2^/day as a 30-min infusion) and 5-fluorouracil (600 mg/m^2^/day as a 120-h continuous infusion), administered on days 1–5 and 29–33 of RT. The maximum daily dose was 1800 mg.
- *Concomitant definitive chemotherapy with hyperfractionated-accelerated RT:*
  Cisplatin (20 mg/m^2^/day as a 30-min infusion) administered on days 1–5 and 29–33 of RT and paclitaxel (25 mg/m^2^/day as a 30-min infusion) twice a week during the course of RT
- *Concomitant palliative chemotherapy:*
  Mitomycin-C (10 mg/m^2^/day as a 30-min infusion) administered on day 1 and, if necessary, on day 29 during RT. The maximum daily dose was 18 mg [23].

### Oral treatment prior to RT

From 2003 onwards, almost all patients were referred to the Department of Dental, Oral and Maxillofacial Medicine for control of the dental infectious source prior to RT.

Dental treatment was performed based on the recommendation “Dental treatment of patients undergoing head and neck cancer radiotherapy” of the German Society for Dental and Oral Medicine [24]. The initial clinical examination was performed by a dental assistant of the University Clinic of Prosthodontics. In coordination with a medical or dental assistant at the University Clinic of Oral and Maxillofacial surgery and considering the clinical and radiological findings, the extent of treatment was determined [4, 5, 25]. All dentulous patients received custom-made fluoride carriers of 5 mm-thick ethylene vinyl acetate [4].

### Evaluation of ORN

Diagnosis and surgical therapy of advanced ORN was performed by a specialist of oral and maxillofacial surgery at the University Clinic Halle-Wittenberg. The advanced ORN stage was classified as grade 3, 4, or 5 according to the Radiation Therapy Oncology Group/European Organization for Research and Treatment of Cancer classification or as grade 3 or 4 according to the late effects in normal tissue/subjective, objective, management, and analytic scale. All tumor stages were ≥ II according to Schwartz and Kagan, stage 3 according to Store and Boysen, and grade 3, 4, or 5 according to Glanzmann and Gratz [14, 26, 27].

### Statistical analysis

Patients were included into the study from January 1, 2003, through January 31, 2013. The last date of follow-up was November 15, 2013, when the study was terminated. The start date was the first day of RT. Data on patients were censored at the termination of the study, on the last date of contact for patients lost to follow-up, or on the date of death. The date of incident ORN was defined as the day of diagnosis.

Since ORN can occur at any time after RT and the five-year survival rate of patients with head and neck cancer is specified as 50 %, it seemed appropriate to consider the five-year probability of survival in the analysis of ORN risk [28, 29].

To include disease-related mortality in the determination of patients’ ORN risk, the cumulative incidence (R) was calculated using the exponential formula [30].

The associations of sex, tumor site, bone surgery during tumor resection, chemotherapy, and dentition with ORN risk was assessed using Cox proportional hazards regression [31]. Dentition was dichotomized as dentulous or edentulous. We estimated the adjusted hazard ratios (HRs) and corresponding 95 % confidence intervals. All variables were mutually adjusted. Analyses were performed with IBM SPSS Statistics 22 (IBM Incorp., SPSS Inc., Chicago, IL, USA) and SAS 9.3 (SAS Incorp., Cary, NC, USA).

## Results

### Patient characteristics

The analysis included 776 patients. On the basis of anatomical region and the resultant target volume, most of the patients had tumors in the oral cavity. Only a few patients had tumors in the uvula. Nearly half of the patients (47 %) were treated with concomitant chemotherapy. About one-eighth underwent bone surgery during tumor resection prior to RT. A detailed breakdown of data concerning tumor site, age, sex, concomitant chemotherapy, and bone surgery is provided in Table 1.

**Table 1:** Distribution of sex, age, concomitant chemotherapy and bone surgery with regard to tumour site

Tumour site	Number of patients (proportion of the cohort)	Proportion of females	Average age in years (standard deviation, range)	Chemotherapy (proportion of tumour localization)	Bone surgery (proportion of tumour localization)
Nasopharynx	43 (5.5 %)	37.2 %	55.9 (±16.4, 22–90)	31 (72 %)	0
Tonsil	157 (20.2 %)	24.2 %	57.6 (±10.5, 24–84)	76 (48 %)	9 (6 %)
Uvula	6 (0.8 %)	16.7 %	67.6 (±8.1, 63–76)	1 (17 %)	0
Tongue base	63 (8.1 %)	22.2 %	58.4 (±10.5, 39–82)	34 (54 %)	4 (6 %)
Oral cavity	259 (33.4 %)	21.6 %	58.9 (±12.1, 21–89)	107 (41 %)	73 (28 %)
Parotid gland	34 (4.4 %)	40 %	62.4 (±13.6, 27–84)	6 (18 %)	3 (9 %)
Hypopharynx/larynx	214 (27.6 %)	11.3 %	59.2 (±9.7, 37–86)	110 (51 %)	1 (0.5 %)

The average patient age was 58.6 ± 11.4 years. At the time of RT, the youngest patient was 21 years and the oldest patient was 90 years old. Seventy-nine percent of all patients were male. Most of the patients had an advanced clinical tumor stage. At the end of the study, 45.9 % of the patients were alive (Table 2).

**Table 2:** Overview about the key figures of the cohort

Sex	♂ *N* = 613	♀ *N* = 163
Five-year probability of survival	44.1 ± 2.1 %	55,5 ± 4.1 %
Average age in years (standard deviation, range)	58.1 (±10.6, 21–89)	60.4 (±14.1, 24–90)
Clinical stage according to UICC		
I	5.2 %	8.6 %
II	9.5 %	9.8 %
III	16.0 %	19.6 %
IV a	56.1 %	50.9 %
IV b	4.6 %	3.7 %
IV c	4.7 %	2.5 %
Unknown	3.9 %	4.9 %

*Abbreviations*: *UICC* Union internationale contre le cancer

### ORN

Of the 776 patients, 51 developed advanced ORN that required extensive surgical intervention (Table 3). About 78 % of patients were male, with an age average of 55.2 ± 10.1 years. The majority of the patients (67 %) had tumors in the oral cavity. By contrast, no patient with a tumor in the nasopharynx developed ORN. Almost half of these patients underwent bone surgery during surgical tumor resection prior to RT. Most patients with tumors in the oral cavity underwent marginal resection of the mandible. Fifty-one percent received concomitant chemotherapy. Of the 51 patients who developed ORN, 42 were treated with 3D-CRT and 9 with IMRT. The median latency period was 9 months (range, 0–90 months).

**Table 3:** Characteristics of the ORN patients with regard to tumor site

Parameters	Tongue base	Parotid gland	Tonsil	Hypopharynx/larynx	Oral cavity	Total
Patients (*N*)	3	2	9	3	34	51
Proportion of males						78 %
Average Age						55.2 ± 10.1
IMRT	1	1	1	0	6	9 (17.6 %)
Mean dose (range)	66.6 Gy (64–72 Gy)	70 Gy (70Gy)	67.23 Gy (64–72Gy)	67.53 Gy (64–69.6 Gy)	66.55 Gy (59.4–72.8 Gy)^a^	66.76 Gy (59.4–72.8 Gy)^a^
Chemo	3	1	4	3	15	26 (50.9 %)
T stage						
T1			1		8	9 (17.6 %)
T2	1	1	4	2	12	20 (39.2 %)
T3	1		2	1	1	5 (9.8 %)
T4	1	1	2		13	17 (33.3 %)
N stage						
N0		1	2	1	12	16 (31.4 %)
N1			2		10	12 (23.5 %)
N2	3	1	5	2	10	21 (41.1 %)
N3					2	2 (2.9 %)
Bone surgery	1	1	4	0	20	26 (50.9 %)
Periostal resection	0	0	0	0	2	2 (3.9 %)
Marginal resection	0	1	1	0	10	12 (23.5 %)
Segmental resection	0	0	1	0	5	6 (11.8 %)
Temporary splitting	1	0	2	0	3	6 (11.8 %)

^a^2 patients received second RT (50 Gy) due to disease's recurrence”

The cumulative incidence of ORN development was 12.4 %. For the multivariate Cox model, the hypopharynx/larynx group was chosen as reference for the tumor site analysis. The estimated HRs and 95 % confidence intervals are presented in Table 4. The oral cavity as the tumor site and bone surgery had the highest values.

**Table 4:** Hazard ratios of the different variables calculated with multivariate analysis

Variable	Hazard ratio	95 % confidence interval	CLR	*p*-value
sex	0.89	0.42–1.90	4.5	0.76
Chemotherapy	1.19	0.67–2.14	3.2	0.55
Dentition (dentulous vs. edentulous)	1.76	0.93–3.33	3.6	0.08
Oropharynx	2.76	0.73–10.38	14.2	0.13
Tongue base	1.94	0.32–11.67	36.5	0.47
Oral cavity	4.69	1.33–16.52	12.4	0.02
Parotid gland	3.04	0.48–19.23	40.1	0.24
Bone surgery	5.87	3.09–11.19	3.6	<.0001

## Discussion

Of the 776 patients included in this study, 51 developed advanced ORN. Based on this number and the total size of the cohort, the relative frequency was 6.6 %. This value was in the upper range of the reported relative frequency of 0 to 7.1 % in a previous study [32]. Based on tumor site, the highest relative frequency was calculated for tumors in the oral cavity (13.6 %). The relative frequency was 5.8 % for patients with tumors in the tonsil and 1.4 % for those with tumors in the hypopharynx/larynx. Similar differences in relative frequency according to tumor site have been reported in the literature [32]. Higher relative frequencies were found in studies primarily evaluating patients with new malignant formations within the oral cavity. Conversely, investigations examining patients with larynx carcinoma usually reported lower relative frequencies [32].

Although most previous studies have used relative frequency as the parameter to evaluate ORN risk, risk assessment by this method can be imprecise. For example, important aspects such as the disease-specific survival rate or the finiteness of the observation period are completely disregarded in the calculation of the relative frequency.

Calculation of the cumulative incidence takes into considerations these aspects in the ORN risk analysis [30]. Thus, in this study, considering the deaths in the cohort, the cumulative incidence was 12.4 %, compared to the relative frequency of 6.6 %. This suggest that the real risk for ORN development lasts a lifetime and may increase by a factor of two in long-time survivors, compared to patients who die early.

The studies published to date have evaluated risk factors based on relative frequency. However, the average survival rate of nearly 50 % in cases of head and neck cancer has not been considered in these calculations of relative frequency. Moreover, the extent to which variables that might influence ORN development influence each other has not been considered. For a more precise assessment, the influence of potential variables was calculated using Cox regression in this study.

Sex was evaluated as a potential variable, because Reuther et al. showed a three times lower risk for ORN in women. They suspected a less frequent consumption of nicotine and alcohol was responsible for this finding [10]. In the current study, the relative frequency of ORN was slightly higher in males (6.7 %) than in females (6.1 %). On additionally considering the factor of survival, HRs revealed a reverse trend. Women were considerably more likely to develop ORN because of their longer survival. Thus, the lower risk of developing ORN in women, as shown by Reuther et al., could not be proved [10]. Hence, whether there is a real difference in nicotine and alcohol consumption between men and women and whether sex is suitable as a surrogate are still unclear. In the participating clinics, nicotine and alcohol consumption were recorded based on patient recall by interviews during examination at admission. Thus, answers may have been biased due to social norms, particularly if questions about frequency of consumption were posed. Moreover, a high discrepancy was found between the statements given in the different clinics. Therefore, this patient-derived information was not included in the calculation model. Accordingly, whether nicotine and alcohol consumption can influence ORN development could not be determined in this study.

Concomitant chemotherapy did not have a clinically relevant influence on the development of ORN (HR = 1.19, CLR = 3.2). Although the degree of precision of this estimate emphasizes the statistical power of the information, it is not possible to clarify whether this result is applicable to all chemotherapeutics used in the treatment of head and neck cancer.

The variables that highly influenced the development of ORN were the performance of bone surgery during tumor resection (HR = 5.87, CLR = 3.6) and the oral cavity as the tumor site (HR = 4.69, CLR = 12.4).

The tumor site represents the anatomical localization of the irradiation volume. In patients with tumors in the oral cavity, the mandible was at least partially included in the primary PTV and received a high (not less than 60 Gy) therapeutic dose. After bone surgery, the already operated on arch segments, e.g., in cases of squamous cell carcinoma with jaw bone infiltration (pT4), the former tumor bed within the primary PTV received the highest prescribed dose (between 64 and 66 Gy). It was therefore not surprising that these patients had, compared to patients without bone surgery, a considerably higher risk of developing ORN. According to the results of Studer et al. the majority of the patients who developed ORN underwent marginal resection of the mandible prior to RT [17].

Cox regression analysis revealed that existing teeth only played a tangential role in the development of ORN (HR = 1.76, CLR = 3.6). Without sufficient irradiation-induced fibrous/atrophic damage of the tissue, the comparatively less extensive extraction trauma does not seem to induce profound damage to the jaw bone. The studies of Studer et al. as well as those of Morrish et al., and Lee et al., confirm this hypothesis [14, 33, 34]. However, the precision of this estimate supports the assumption that dentition might become a relevant factor in patients who do not have to undergo bone surgery and do not have head and neck cancer within the oral cavity.

Regarding these results, patients with tumors in the oral cavity who had to undergo bone surgery prior to RT seem to be a small high-risk patient group for the development of ORN. These findings are confirmed by the results of Parliament et al. who showed that the mean dose found in the mandible using IMRT is higher in oral cancer than in other tumor sites [35]. Even using advanced planning technqiues like IMRT it remains possible to have higher irradiations dose to the mandible. Identifying this group as a high-risk group offers the possibility to pay particular attention to these patients and to take prophylactic measures.

## Conclusions

Considering survival probability, the cumulative incidence of ORN was 12.4 %. Bone surgery during tumor resection and the oral cavity as the tumor site were associated with the highest risk of ORN development. In contrast, the nasopharynx and hypopharynx/larynx as tumor sites were associated with a very low risk. In addition, sex and concomitant chemotherapy showed no verifiable influence. When adequate dental treatment was performed prior to RT, no influence of the remaining teeth on the development of ORN could be verified.

## Abbreviations

3D CRT, 3 dimensional radiotherapy; CI, confidential interval; CRT, conformal radiotherap; Gy, gray; HR, hazard ratio; IMRT, intensity modulated radiotherapy; ORN, osteoradionecrosis; RT, radiotherapy
